# Comparing 3D foot scanning with conventional measurement methods

**DOI:** 10.1186/s13047-014-0044-7

**Published:** 2014-10-25

**Authors:** Yu-Chi Lee, Gloria Lin, Mao-Jiun J Wang

**Affiliations:** Department of Industrial Engineering and Engineering Management, National Tsing Hua University, No. 101, Section 2, Kuang-Fu Road, Hsinchu, 30013 Taiwan

## Abstract

**Background:**

Foot dimension information on different user groups is important for footwear design and clinical applications. Foot dimension data collected using different measurement methods presents accuracy problems. This study compared the precision and accuracy of the 3D foot scanning method with conventional foot dimension measurement methods including the digital caliper, ink footprint and digital footprint.

**Methods:**

Six commonly used foot dimensions, i.e. foot length, ball of foot length, outside ball of foot length, foot breadth diagonal, foot breadth horizontal and heel breadth were measured from 130 males and females using four foot measurement methods. Two-way ANOVA was performed to evaluate the sex and method effect on the measured foot dimensions. In addition, the mean absolute difference values and intra-class correlation coefficients (ICCs) were used for precision and accuracy evaluation. The results were also compared with the ISO 20685 criteria.

**Results:**

The participant’s sex and the measurement method were found (*p* < 0.05) to exert significant effects on the measured six foot dimensions. The precision of the 3D scanning measurement method with mean absolute difference values between 0.73 to 1.50 mm showed the best performance among the four measurement methods. The 3D scanning measurements showed better measurement accuracy performance than the other methods (mean absolute difference was 0.6 to 4.3 mm), except for measuring outside ball of foot length and foot breadth horizontal. The ICCs for all six foot dimension measurements among the four measurement methods were within the 0.61 to 0.98 range.

**Conclusions:**

Overall, the 3D foot scanner is recommended for collecting foot anthropometric data because it has relatively higher precision, accuracy and robustness. This finding suggests that when comparing foot anthropometric data among different references, it is important to consider the differences caused by the different measurement methods.

## Background

Foot dimension measurements are important for footwear design, fit evaluation and clinical applications [1-3]. Wearing footwear that does not fit an individual’s foot characteristics may increase the risk of having lower extremity musculoskeletal problems including foot pain and deformity [4]. Designing footwear using foot size and shape information will enhance the fit of the shoes [1,2,5,6]. Different measurement methods have been applied to collect foot characteristic information such as dimensions, foot shape and plantar contour. The most common approaches include using digital calipers for direct measurements and 3D scanning and footprint analysis for indirect measurements.

The ink footprint and digital caliper are the traditional manual approaches used to collect foot dimensions. However, the accuracy of digital caliper measurement tends to be affected by human error [7,8]. Different technicians may obtain inconsistent measurement results [7]. It is very important to provide adequate training for the technicians to correctly position landmarks on the proper anatomical points [8]. Using the ink footprint to collect foot dimensions can reduce the experiment time. The ink footprint data can also be used repeatedly for different applications such as calculating the arch index at a convenient time. However, the limitation of collecting foot dimensions using ink footprint method is that it cannot measure vertical dimensions such as navicular height. In addition, the quality of the ink footprint may influence the precision and accuracy of foot dimension measurements.

A 3D scanning method developed through advanced optoelectronic technologies has been employed to collect anthropometric data [9,10]. The 3D scanning technique can obtain human body surface, volume and cross sectional information. Advantages of using 3D foot scanning are that it allows a large number of participants to be scanned quickly and the measurement is robust and efficient [11]. The disadvantage is the high initial set-up cost. The 3D foot scanning images can also be used to obtain digital footprints. Previous study has indicated that using the digital footprint to collect foot dimensions is reliable [12].

A number of studies have been conducted to compare the measurement differences of using different instrumentations and methods. For example, Witana et al. [13] compared the caliper, 3D scanning and a new automated approach to collect foot dimensions from 3D scanning images and indicated that 8 out of 18 foot dimensions showed significant differences among the three methods. Mall et al. [14] compared the foot dimensions collected using optical techniques and caliper measurements and reported that using the optical techniques was as reliable as the caliper measurements and the measurement time was reduced. Zhao et al. [15] proposed an approach to obtain 6 foot girths to customize footwear and the results indicated that there were less than 5 mm differences in measures when compared with the manual measurements. De Mits et al. [16] conducted a study to evaluate the validity of 3D scanning measurements using comparisons with X-ray and manual measurements. They indicated that the 3D scanning method showed good validity when scanning healthy participants. For abnormal feet, the 3D scanning method can also be applied to screen for foot deformities before the presence of erosions [17] and it has demonstrated good validity and reliability compared with clinical measurements [18]. Noldner and Edgar [19] compared 3D, 2D and manual measurements in describing morphology and reported that the 3D scanning method was encouraging.

It appears that using different methods to collect foot dimensions may lead to inconsistent results. However, comparison information on the most commonly used foot measurement methods is currently lacking. This study compared the precision and accuracy of the four most common foot measurement methods (i.e. digital caliper, 3D scanning, digital footprint and conventional footprint measurements).

## Methods

### Participants

One hundred and thirty participants (65 males and 65 females) were recruited in this study. The mean age of the males was 21.25 ± 2.15 years, ranging from 18 to 28 years. Their mean height and weight was 174.92 ± 5.82 cm and 68.45 ± 7.57 kg, respectively. The mean age for females was 21.98 ± 2.94 years, ranging from 18 to 30 years. Their mean height and weight was 162.09 ± 4.30 cm and 52.32 ± 5.89 kg, respectively. The participants were undergraduate and graduate students from a university in Taiwan. All participants were healthy and right-handed. Participants were requested to be free of musculoskeletal disorders and not experiencing any pain or medical conditions affecting their feet, ankles, or lower back. The experimenter measured only the dominant foot because of the time constraint. The dominant foot was defined as the one most vigorously used in activities, for example kicking a ball [20-22] and was determined by a self-reporting procedure. The right foot was dominant for all participants. All measurements were taken in the late morning to avoid foot volume deformation. The protocol for this study was approved by the institutional ethics committee of National Tsing Hua University (reference number 10306HE023).

### Foot dimensions and landmarks

Six foot dimensions, including foot length, ball of foot length, outside ball of foot length, foot breadth diagonal, foot breadth horizontal and heel breadth were measured using the four foot measurement methods (i.e. digital caliper, 3D foot scanner, digital footprint and ink footprint). The definitions of the foot dimensions are presented in Table 1. The selected six foot dimensions are relevant to shoe and insole design.

**Table 1:** **Definition of the six foot dimensions**

**Dimensions**	**Definition**	
1. Foot length	The direct distance from pternion point to the most anterior point of the longest toe (first or second) measured parallel to the foot axis.	
2. Ball of foot length	The distance from the end of heel to the metatarsal tibial measured parallel to the foot axis.	
3. Outside ball of foot length	The distance from the end of heel to the metatarsal fibular measured parallel to the foot axis.	
4. Foot breadth diagonal	The distance between the metatarsal tibial and metatarsal fibular of the ball cross section projected to the standing surface.	
5. Foot breadth horizontal	The horizontal distance between metatarsal tibial to metatarsal fibular.	
6. Heel breadth	The breadth of position at 16% foot length straight from the pternion point to toe.	

Foot axis: The line passes from the pternion to the tip of the second toe.

Before data collection, a well-trained experimenter placed 2 markers on the landmark positions of the participant’s right foot surface. The marker was a 4.2 mm blue colored sticker. The two landmark positions were the metatarsal tibial and metatarsal fibular. The metatarsal tibial was defined as the most medial point on the head of the first metatarsal of the foot. The metatarsal fibular definition was the most lateral point on the head of the fifth metatarsal. The same two anatomical points were used for digital caliper measurement and 3D scanning measurement. The rearmost point of the heel was defined as the pternion. The definition of each of the foot dimensions was consistent among the four foot measurement methods. For example, the heel breadth was defined as the breadth of position at 16% of the foot length (FL) in a straight line from the pternion point to the toe. The XY plane is the standing surface for the foot coordinate system. The origin is the pternion projected on the XY plane. The X axis is identical with the foot axis which is the line passing from the pternion to the tip of the second toe. The medial direction is Y+, and the vertical direction is Z+. The same coordinate system was used for 3D scanning, digital caliper, ink footprint and digital footprint measurements to avoid the different alignment method effect.

### Four different measurement methods

**Figure 1:**
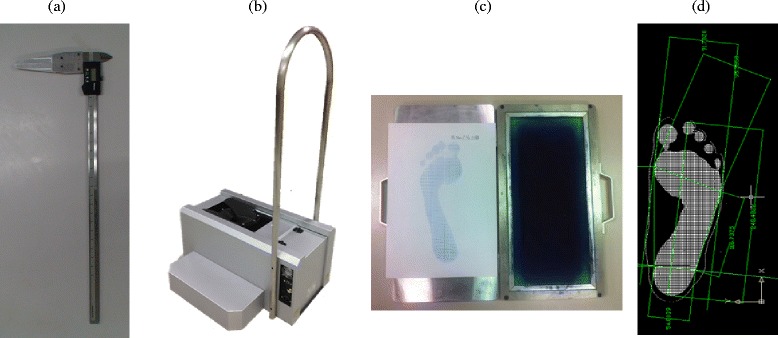
**The four measurement methods: digital caliper (a), 3D foot scanner (b), Harris mat (c) and digital footprint image (d).**

The four measurement methods used in this study are illustrated in Figure 1. A trained experimenter conducted the digital caliper measurements (Mitutoyo Corp., Tokyo, Japan), measuring the foot dimensions based on the two specified anatomical landmarks. A sliding caliper with nib style jaws was used for measurements. The measurement range was from 0.5 mm to 500 mm and its resolution was 0.01 mm.

A 3D foot scanner (INFOOT USB scanning system, IFU-S-01, I-Ware Laboratory Co., Ltd, Japan) was used to collect the foot dimensions. The 3D foot scanner has 8 CCD cameras and 4 laser projectors to construct the foot structure. Cross sectional data were measured at 1.0 mm intervals over the entire foot surface including the sole. The foot scanner resolution was within 0.1 mm. The anatomical landmark coordinates were measured using the two markers pasted onto the foot surface. After automatic detection and recognition of the landmarks with the INFOOT system, 6 foot dimensions were collected.

The Harris mat (force foot imprinter kit, ACCS-00167, Acor Orthopaedic, Inc., USA) was used for the ink footprint measurement. A 3D foot model obtained from the 3D foot scanner was used to collect the contour of the plantar surface as the digital footprint. The 3D foot scanning model based on triangular meshes was used to collect the digital footprint [23]. To avoid noise influence the contour at 1.5 mm height from the plantar surface of the foot was used as the digital footprint. All digital footprint images were first stored in a PC. The AutoCAD software package was used to collect the six foot dimensions from the digital footprint by calculating point to point distances. The software was calibrated to the image dimensions before use. The digital footprint image lines were drawn directly onto the image to obtain the specified foot dimensions. The experimenter can zoom in the digital footprint image to identify the most outermost point of the foot (i.e. metatarsal tibial and metatarsal fibular point) for collecting width dimensions. Six feature points were identified, including the tip of the longest toe (first or second), metatarsal tibial point, metatarsal fibular point, medial malleolus point, lateral malleolus point and pternion point to conduct the foot measurements. All of the points were identified in the digital footprint by one experimenter.

### Data collection and procedure

The dominant foot of each participant was measured using three foot measurement methods. The digital footprint was collected after the foot scan image. The sequence for the three foot dimension measurement methods was assigned randomly to each participant. All three trials were measured on the same day. The two landmarks were not removed until all of the measurements were taken including the repeated measurements using different foot measurement methods. To assure data collection accuracy and consistency only one well-trained experimenter was involved in positioning the landmarks and conducting manual foot measurements.

Each participant was asked to stand on the marked floor with a normal upright posture and align his/her pternion point along a horizontal guiding line and toe 2 with a vertical guiding line in a 2D coordinate system. The participants were requested to keep their two feet separated shoulder width apart to ensure that their body weight was equally distributed onto both feet before data collection. After one successful foot dimension measurement, the participant was asked to take a short break. The same procedure was followed for the next measurement.

Each participant was requested to wash his/her right foot and use tissue paper to dry the foot surface completely before scanning. This procedure was to avoid measurement errors due to particles adhered to the foot surface. The participants then stood on a glass plate and positioned their right foot in the scanner using a stable standing posture, avoiding any foot movement. After a successful scan the right foot was removed from the scanner. The experimenter then used glass cleaner to clean the dust and prints from the standing glass plate. The same procedure was then repeated for the second scan. This routine was used to avoid operator bias and ensure scanning quality. The foot scanning took about 1 minute with two repeated measurements taken. The experimenter checked all foot scanning images and manually removed the noise when necessary after each scan. Any obvious landmark position errors were corrected at that time.

The participant was requested to stand naturally on a Harris mat for the ink footprint measurement. The participants were asked to distribute their body weight equally over both feet. The right footprint was then inked on a paper surface. The foot was then removed from the Harris mat. The experimenter removed the footprint paper and replaced it with a new one for the next foot measurement. The ink print quality of the toes and outside foot contour was ensured for accurate data collection. Each participant was measured twice using the same procedure. The ink footprint contour was drawn on paper. The experimenter identified the metatarsal tibial and fibular points and measured the six foot dimensions from the ink footprint.

### Data analysis

The independent variables were sex (male and female) and foot measurement method (caliper measurement, 3D scanning, digital footprint and ink footprint method). The dependent variables were the six foot dimensions. Two-way analysis of variance (ANOVA) was conducted to evaluate the sex and method effect on the collected six foot dimensions. Duncan’s multiple range test (MRT) was employed for post hoc comparison on the significant factors. Intra-class correlation coefficients (ICCs) for the four measurement methods were calculated for comparison. All of the statistical analyses were performed using the SPSS 18.0 software package. The significance level was set at α = 0.05.

The mean absolute difference (MAD) between the repeated measurements for the four methods was defined as the precision (repeatability) measure for this study. MAD was commonly used to evaluate the precision of the measurement method [7,9,24]. A smaller MAD value indicates higher precision. ISO 20685 [25] provided maximum allowable difference as the performance index for accuracy [23,24,26]. Following ISO 20685 the accuracy level of 2 mm was considered as the standard requirement when taking foot measurements. The accuracy was defined as the extent to which the measured value approximated the true value. The digital caliper measurement was considered as the true value due to its relatively high instrument resolution (0.01 mm).

## Results

The ANOVA results are presented in Table 2. Both factors (sex and measurement method) showed significant effects on the six foot dimensions. No significant interaction effect was found in the selected measures.

**Table 2:** **Summary of two-way ANOVA results**

**Term**	**d. f.**	**Foot length**	**Ball of foot length**	**Outside ball of foot length**	**Foot breadth diagonal**	**Foot breadth horizontal**	**Heel breadth**
Sex	1	***	***	***	***	***	***
Method	3	***	***	***	***	***	***
Sex* Method	3	NS	NS	NS	NS	NS	NS

***Significant difference (p < 0.001); NS: no significant difference; d.f.: degree of freedom.

**Figure 2:**
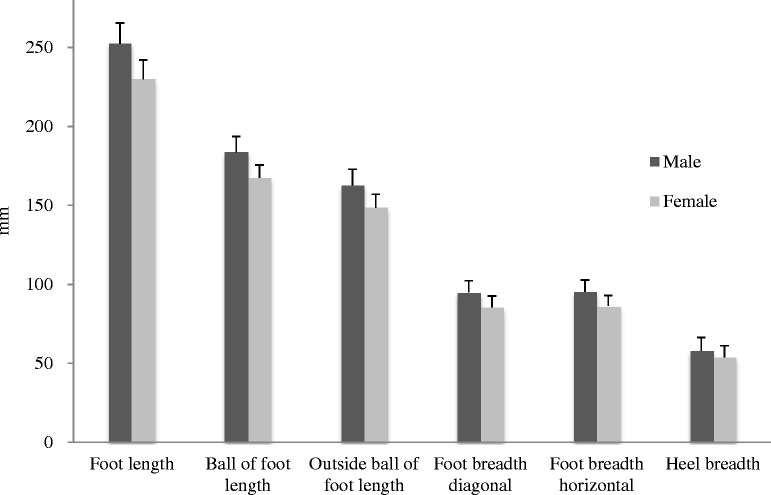
**The sex differences in six foot dimensions among measuring methods (unit in mm).**

Males had significantly greater foot dimensions than females regardless of the measurement method. The differences between the sexes were about 22.4 mm (range 21.2 to 23.2 mm) in foot length, 16.1 mm (range 15.1 to 17.6 mm) in ball of foot length, 14.1 mm (range 12.1 to 15.6 mm) in outside ball of foot length, 9.4 mm (range 8.1 to 10.7 mm) in foot breadth diagonal, 8.9 mm (range 8.1 to 10.4 mm) in foot breadth horizontal and 4.4 mm (range 3.6 to 5.7 mm) in heel breadth dimension among the four different measurement methods (as shown in Figure 2).

Table 3 displays the Duncan’s multiple range test results for the six foot dimensions regardless of sex. Significant differences were found in selected measures in the four foot measurement methods. The six foot dimensions obtained from the 3D scanning method had greater values than the others. No significant difference was found between the digital footprint and ink footprint measurements, except for the outside ball of foot length and heel breadth. Foot dimensions obtained using the digital footprint and ink footprint methods exhibited smaller measurement values in five of the six dimensions than those obtained using 3D scanning and the digital caliper.

**Table 3:** **ANOVA and Duncan’s MRT results among the four methods**

**Dimension**	**Foot length (mm)**		**Ball of foot length (mm)**		**Outside ball of foot length (mm)**		**Foot breadth diagonal (mm)**		**Foot breadth horizontal (mm)**		**Heel breadth (mm)**	
	**Mean (SD)**	**Duncan’s MRT**	**Mean (SD)**	**Duncan’s MRT**	**Mean (SD)**	**Duncan’s MRT**	**Mean (SD)**	**Duncan’s MRT**	**Mean (SD)**	**Duncan’s MRT**	**Mean (SD)**	**Duncan’s MRT**
3D scanning	249.3 (15.7)	A	181.6 (11.6)	A	164.1 (10.64)	A	99.0 (7.3)	A	97.1 (7.0)	A	63.2 (4.5)	A
Digital caliper	247.7 (15.7)	A	177.7 (12.7)	B	157.0 (10.8)	B	94.7 (7.2)	B	85.2 (7.4)	B	62.6 (4.0)	A
Digital footprint	233.0 (14.8)	B	170.6 (10.9)	C	153.4 (9.9)	C	87.8 (6.4)	C	86.0 (6.3)	B	50.1 (3.8)	B
Ink footprint	234.1 (14.6)	B	172.0 (10.6)	C	147.7 (9.3)	D	88.6 (6.5)	C	85.1 (6.2)	B	47.7 (3.9)	C
p-value	p < 0.001		p < 0.001		p < 0.001		p < 0.001		p < 0.001		p < 0.001	

For precision evaluation, all MAD values for the four measurement methods were less than 3 mm (Table 4). The 3D scanning method had higher precision performance than the other three methods in four of the six foot dimensions (i.e. ball of foot length, outside ball of foot length, foot breadth diagonal and foot breadth horizontal dimensions). The digital caliper method had higher precision performance than the others in two of the six foot dimensions (i.e. foot length and heel breadth). The digital caliper method had lower precision performance than the others in the ball of foot length and outside ball of foot length. The ink footprint method had the worst precision performance in measuring foot dimensions, except for ball of foot length and outside ball of foot length.

**Table 4:** **Precision evaluation for the four methods**

**Dimension**	**MAD** ^**a**^ **(mm)**				**Maximum allowable error in ANSUR [** 27 **] (mm)**
	**3D foot scanning**	**Digital caliper**	**Digital footprint**	**Ink footprint**	
Foot length	1.50	1.41	1.53	1.60	3.00
Ball of foot length	1.36	2.40	1.46	1.80	6.00
Outside ball of foot length	1.34	2.01	1.41	1.90	
Foot breadth diagonal	0.73	1.42	0.77	2.65	
Foot breadth horizontal	0.79	1.66	0.83	1.75	2.00
Heel breadth	0.91	0.80	0.86	1.20	2.00

^a^MAD between the repeated measurements.

The accuracy evaluation results are summarized in Table 5. The MADs of the 3D scanning measurements were less than 11.9 mm in six foot dimensions. The 3D scanning measurements had the smallest MADs than the other methods, except for outside ball of foot length and foot breadth horizontal. By comparing the results of this study with the ISO 20685 criteria, the MAD for foot length and heel breadth in 3D scanning measurement met the maximum allowable difference. The digital footprint and ink footprint measurement accuracy was not satisfactory, except for foot breadth horizontal. The ball of foot length, outside ball of foot length, foot breadth diagonal and foot breadth horizontal collected from 3D scanning measurements were not accurate enough for the anthropometric database.

**Table 5:** **Accuracy evaluation for the four methods**

**Dimensions**	**MAD** ^**a**^ **(mm)**			**ISO 20685 [** 25] **(mm)**
	**3D scanning**	**Digital footprint**	**Ink footprint**	
Foot length	1.6	14.7	13.6	2.00
Ball of foot length	3.9	7.1	5.7	2.00
Outside ball of foot length	7.1	3.6	9.3	2.00
Foot breadth diagonal	4.3	6.9	6.1	2.00
Foot breadth horizontal	11.9	0.8	0.1	2.00
Heel breadth	0.6	12.5	14.9	2.00

^a^MAD between the digital caliper measurement and the 3D scanning, ink footprint and digital footprint measurement.

The ICCs for all six foot measurements using the digital caliper were within the 0.74 to 0.98 range (as shown in Table 6). The worst reliability was found in the ball of foot length dimension. The ICCs for the scanning measurements and digital footprint measurements were within the 0.95 to 0.98 and 0.94 to 0.98 ranges, respectively. The ICC range for the ink footprint measurements was 0.59 to 0.91 and the worst performance was found in the foot breadth diagonal dimension.

**Table 6:** **The intra-class correlation coefficients (ICC) for the four measurement methods**

**Dimensions**	**ICCs**			
	**3D scanning**	**Digital caliper**	**Digital footprint**	**Ink footprint**
Foot length	0.98	0.98	0.97	0.91
Ball of foot length	0.95	0.74	0.97	0.68
Outside ball of foot length	0.95	0.89	0.94	0.98
Foot breadth diagonal	0.95	0.89	0.98	0.59
Foot breadth horizontal	0.96	0.84	0.98	0.93
Heel breadth	0.96	0.87	0.94	0.78

## Discussion

**Figure 3:**
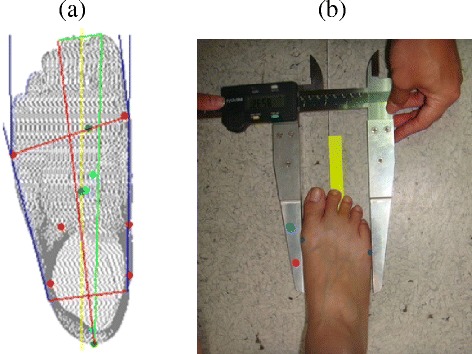
**Measuring foot breadth horizontal dimension by using (a) scanning image and (b) digital caliper.**

The results from this study indicated that the foot dimensions collected from the 3D scanner were greater than the foot dimensions collected from the other three methods. This was because the 3D scanner detects the outermost point of the metatarsal head easier than manual methods when measuring the ball of foot length, outside ball of foot length and the two foot breadth (diagonal and horizontal) dimensions (as shown in Figure 3). The experimenter palpates the metatarsal point protrusion during using the digital caliper method which may not be the outer most point for taking foot dimension measurements. Another reason may be that the experimenter may compress the soft tissue surrounding the landmark while using digital calipers to take measurements. Thus, measurement procedure standardization and adequate training for the measurer should be emphasized.

The 3D foot scanner accuracy was less than the digital caliper approach due to the instrument resolution and landmark position identification limitation. The foot dimensions collected from the digital footprint and ink footprint were smaller than the foot dimensions collected using the digital caliper and 3D foot scanner. This was because the pressure distribution on the edge of the plantar surface was lower and the footprint contour captured tends to be smaller than the actual plantar surface contour. Moreover, the footprint contour was drawn on paper. When drawing the footprint, the soft tissue bulge was not included, leading to foot dimensions underestimation.

The results showed that the 3D foot scanner had the best precision performance among the four methods because less manual effort was involved. The digital caliper approach showed better precision in two of the six foot dimensions (foot length and heel breadth dimensions). This is because the positions for measuring foot length and heel breadth can be easily located by the caliper, and resulting in consistent measurement results. Noise of scanned image, footprint and ink footprint may influence the results between two measurement repetitions, especially in the toe area in which the image is difficult to restructure. These may be the reasons for varying results in this study. On the other hand, the digital caliper method had a greater MAD value in the ball of foot length and outside ball of foot length. The ball of foot length and outside ball of foot length measurements represent a straight line distance from the end point of the heel to the metatarsal tibial and metatarsal fibular, respectively. Since the digital caliper cannot contact the metatarsal tibial (or metatarsal fibular) head and the end point of the heel simultaneously, the extended line of the heel end point was used to take this measurement. This would influence the consistency of the repeated measurements. Moreover, the precision of the ink footprint method appears to be the worst in four of the six foot dimensions among the four methods. This was due to the stability of the plantar surface pressure distribution affecting the ink footprint quality, resulting in poor consistency in the repeated measurements.

The Anthropometric Survey of U.S. Army Personnel (ANSUR) MAD results were applied to examine the maximum allowable error for measurements [9,24,27]. If the MAD value for repeat measurements was smaller than the ANSUR’s maximum allowable error, it indicates that the measurement precision was acceptable. Comparing the MAD values in this study with the maximum allowable error in the ANSUR results, all four measurement methods met the criteria suggesting the precision of the four methods for measuring the six foot dimensions was acceptable.

Table 5 reveals that the two footprint measurements met the ISO 20685 criteria only in foot breadth horizontal. The 3D scanning measurements met the ISO 20685 criteria in two of six foot dimensions. It seems that the 3D scanning measurement was not accurate enough to collect the ball of foot length, outside ball of foot length, foot breadth diagonal and foot breadth horizontal dimensions. Telfer et al. [28] investigated six commonly used methods including 3D scanning measurements for capturing foot shape and reported that none of them can meet all of the standard criteria. The 3D scanning method showed better performance than the two footprint measurement methods in accuracy evaluation in this study. On the other hand, it should be noted that the training and measuring experience of the experimenter might influence digital caliper measurement results. Thus, adequate training of the experimenter is very important.

For the ICC digital caliper measurement results the worst reliability was found in the ball of foot length dimension. This finding was consistent with the report of Mall et al. [14]. Since the first metatarsal head is a rounded and large bone, it is difficult to identify and mark the center position, and thus causing the poor performance in reliability while using a digital caliper to measure ball of foot length. The ICCs of scanning measurements were within the range 0.93 to 0.98, which is similar to the report of De Mits et al. [16] where the ICC of the INFOOT scanning system was 0.94 to 0.99 for measurements of length and breadth dimensions.

The 3D foot scanning method exhibited higher precision and accuracy for collecting foot anthropometric data. The advantages of using the 3D scanning system to collect foot dimensions include shorter measurement time and higher efficiency for measuring a large number samples. The data can be used and reused for different applications at a later time [11]. The 3D foot scanning method can also collect volumetric and surface data and provide more detailed foot size and shape information. However, the disadvantage is that the initial setup cost for the 3D foot scanning system is higher than the other methods. The size of the 3D foot scanning system (INFOOT system) is 685(L) × 400(W) × 310(H) mm and thus the portability of the scanning system is somewhat limited. It is important to note that when using the 3D scanning method to collect foot dimensions, the noise in the 3D image should be checked and removed to improve the precision, particularly in the foot length and heel breadth dimensions.

In order to minimize the influence of human error, adequate training is important for the experimenter to correctly locate landmarks and conduct measurements. Since only one well-trained experimenter was involved in this study the inter-rater repeatability of the four measurements cannot be estimated. This study used only the INFOOT scanning system to obtain 3D foot measurements and the two markers remained on the participants’ foot throughout the measurement process. The differences among different 3D foot scanning systems should be taken into consideration when applying the results of this study.

## Conclusions

This study compared the precision and accuracy of four foot dimension measurement methods. Based on the precision and accuracy evaluation results, applying the 3D scanning method to collect the foot dimensions had better performance than the digital caliper, digital footprint and ink footprint methods. Based on the findings, this study supports the use of 3D scanning method for collecting foot anthropometric data. Moreover, using different instruments to measure foot dimensions may produce inconsistent results. It is important to take into account the measurement method differences when comparing foot anthropometric data.
